# Pulse Intake Improves Nutrient Density among US Adult Consumers

**DOI:** 10.3390/nu13082668

**Published:** 2021-07-31

**Authors:** Diane C. Mitchell, Christopher P. F. Marinangeli, Sandrine Pigat, Foteini Bompola, Jessie Campbell, Yang Pan, Julianne M. Curran, David J. Cai, Susan Y. Jaconis, Jeff Rumney

**Affiliations:** 1 Diet Assessment Center, Department of Nutritional Sciences, The Pennsylvania State University, University Park, PA 16802, USA; 2 Pulse Canada, Winnipeg, MB R3C 0A5, Canada; cmarinangeli@pulsecanada.com (C.P.F.M.); jcurran@pulsecanada.com (J.M.C.); 3 Creme Global Ltd., D02 P956 Dublin, Ireland; sandrine.pigat@cremeglobal.com (S.P.); fwteini_bbl@hotmail.com (F.B.); 4 USA Dry Pea & Lentil Council, American Pulse Association, Moscow, ID 83843, USA; campbellj@msd281.org (J.C.); ejaconis@usapulses.org (S.Y.J.); jrumney@usapulses.org (J.R.); 5 PepsiCo, Inc., Plano, TX 75024, USA; yang.pan2018@gmail.com (Y.P.); davidcai123@gmail.com (D.J.C.)

**Keywords:** pulses, National Health and Examination Survey (NHANES), diet quality, nutrient density, legumes

## Abstract

The objective was to examine trends in pulse (dry beans, dry peas, chickpeas and lentils) intake over a 10-year period and to compare nutrient intakes of pulse consumers and non-consumers to better understand the impact of pulse consumption on diet quality in the US population. NHANES 2003–2014 data for respondents (≥19 years) with 2 days of intake was used to evaluate trends in pulse intake. Pulse consumers were identified as those NHANES respondents who consumed pulses on one or both days. Differences in energy adjusted nutrient intakes between non-consumers and consumers were assessed. There were no significant trends in pulse intakes for the total population or for pulse consumers over the 10-year period. In 2013–2014, approximately 27% of adults consumed pulses with an intake of 70.9 ± 2.5 g/day over 2 days, just slightly <0.5 cup equivalents/day. At all levels of consumption, consumers had higher (*p* < 0.01) energy adjusted intakes of fiber, folate, magnesium. Higher energy adjusted intakes for potassium, zinc, iron and choline and lower intakes of fat were observed for consumers than for non-consumers at intakes ≥69.4 ± 1.01 g/day. These data suggest that pulse consumption in the US population may result in better diet quality with diets that are more nutrient dense than those without pulses.

## 1. Introduction

Pulses, as defined by the Food and Agriculture Organization of the U.N., encompass a narrower class of legumes harvested as a dry grain that includes dry beans, peas, chickpeas and lentils [1]. Other legumes that are harvested while they are still green, contain significant levels of oil (e.g., soybeans and peanuts), or are garden vegetable varieties such as green peas and green beans are not considered pulses. There are hundreds of varieties of pulses grown around the world; however, the most commonly consumed pulses in the U.S. are dry beans (e.g., pinto, black and kidney beans), chickpeas, lentils and dry peas [2].

Legumes, including pulses, have been shown to have many health benefits. Higher intakes of legumes have been associated with satiety, weight management, improved gastrointestinal health, reduced risk of certain types of cancer, cardiovascular disease, hypertension and diabetes [3,4,5,6,7,8,9,10]. Pulses contain phytochemicals or non-nutritive bioactive components, which may have important health benefits [3]. They are a significant source of many nutrients, such as complex carbohydrate, protein, fiber, folate, iron, magnesium, and potassium and are a good source of many other nutrients (e.g., choline, zinc, selenium, phosphorus, and thiamin). For this reason, they are considered a nutrient dense food. Nutrient density is often used to qualify foods based on a given amount, standard serving, or calorie level and is calculated most often by expressing the amount of a specific nutrient per 100 g of food or per 1000 kcal of intake.

The 2020–2025 Dietary Guidelines [11] recommend consuming 1.5 cups of legumes defined as beans and peas per week as part of the vegetable group. However, given their high levels of protein, they can also count as a protein food. Therefore, as a replacement for meat, the relative contribution of pulses to the diet could exceed recommendations set for the vegetable group; however, most often they are consumed in place of other vegetables [2]. Pulses also contain 50–65% carbohydrate including resistance starch, soluble and insoluble fiber, and have a low glycemic index which may be beneficial for blood glucose management [12].

Despite dietary recommendations encouraging consumption of legumes, prevalence estimates from population studies of the National Health and Nutrition Examination Survey (NHANES) data from 1999–2002 showed that less than 8% of Americans were consuming pulses on any given day [2] and there is little evidence that this consumption level has changed since then.

To support dietary guidance that encourages healthy diet patterns with higher intakes of plant-based foods, an updated perspective on pulse consumption and their impact on diet quality is needed. Therefore, the purpose of this study is to update the literature on pulse consumption in U.S. adults by evaluating trends in intake over a 10-year period among pulse consumers and to examine the impact that pulses have on nutrient intakes. These data will contribute to our understanding of the role of pulses in the U.S. diet.

## 2. Materials and Methods

### 2.1. Data Source

Data from NHANES 2003–2014 gathered by the Center for Disease Control, National Center for Health Statistics (NCHS) was the source of data used in the analysis. NHANES is a cross-sectional survey conducted on a continual, annual basis to monitor the health and nutritional status of a nationally representative sample of the U.S. civilian, non-institutionalized population. Details on accessing the data, sampling designs and other methods used are available on the NCHS website [13]. The NCHS Ethics Review Board monitors and approves all survey procedures and written consent is obtained from all survey respondents. Publicly available data are released in 2-year increments as de-identified datasets and, therefore, are exempt from further institutional review board approval. Demographic characteristics and the “What We Eat in America” (WWEIA) or the dietary component of the survey was used to select adult respondents’ age 19–65 years who reported 2 days of dietary intake.

### 2.2. Dietary Data Collection and Analysis

The dietary component of the NHANES survey consists of 2 days of intake collected using USDA’s Automated Multiple Pass Method (AMPM) [14]. The first dietary intake data is collected in a Mobile Examination Center as an in-person interview. The second day of dietary data is collected by telephone within 2 weeks. The average of two-day dietary intakes was used to assess nutrient intakes and the quantity of pulses consumed. Pulse consumers were identified as those respondents who had reported consuming pulses at least once in the 2 days of reported intake. Non-consumers were identified as those who did not consume pulses an either of the 2 days of intake. Dietary intake files containing individual food level data and food codes were used in combination with the Food and Nutrient Database for Dietary Studies (FNDDS) recipe files to quantify the amount of pulses contained in 72 FNDDS food codes. Recipe files contain the amount of pulses in 100 g portions of all pulse containing foods and allows for the quantification of pulses consumed in a variety of mixed or combination foods. To examine nutrient intakes and amount of pulses consumed were derived using FNDDS versions 2003–2004 to 2013–2014 corresponding to each of the 6 cycles of survey used in this study.

### 2.3. Statistical Analysis

All data were analyzed in accordance to the NHANES analytical guidelines using the appropriate survey weights designed to account for unequal selection probabilities, clustered design and non-response. All analyses were conducted by Creme Global (Dublin, Ireland) using the Creme Nutrition^®^ model. Creme Nutrition^®^ is a scientific cloud-based software service used to assess and predict dietary intakes of foods and nutrients in populations of consumers [15]. In Creme Nutrition^®^, standard errors of statistics are calculated using bootstrapping, a resampling technique.

Six 2-year cycles of NHANES 2003 to 2014 were used to examine the trend of pulse consumption over a 10-year period. A linear regression model was applied over the weighted averages of the amount consumed to determine if there were statistically significant trends in pulse consumption in pulse consumers compared to the total population of adults (≥19 years).

Significant differences in demographic characteristics between non-consumers of pulses compared to pulse-consumers were determined by a chi-square test. To test the hypothesis that the energy adjusted intake of nutrients is different between non-consumers and each quartile of pulse consumers, a paired Wilcoxon test was conducted with *p*-values at <0.01 considered to be statistically significant.

## 3. Results

**Figure 1:**
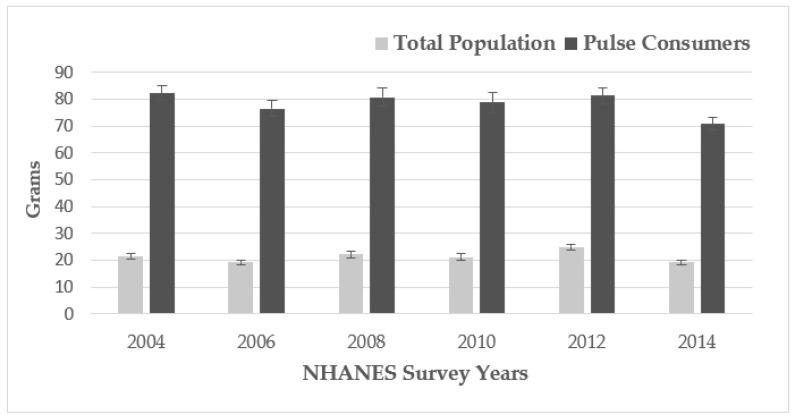
Pulse intake (grams) in the total population vs. pulse consumers, adults ≥ 19 years of age, National Health and Nutrition Examination Survey (NHANES) 2013–2014.

There was no change (*p* = 0.812) over time in per capita consumption of pulses (i.e., average amount of pulses consumed on any given day based on 2-day average intakes) by adults’ age 19 to 65 years across 6 cycles of NHANES from 2003-4 to 2013-14 (Figure 1).

For the total population, per capita daily intakes were the highest in 2011-12 at 24.9 (±1.2) g/day and the lowest intakes were observed in 2006 (19.3 ± 0.9) and 2014 (19.3 ± 0.9) g/day consumed. Based on the average across 2 days, for pulse consumers, daily intakes of pulses were 82.2 (±2.7 g) in 2003-4 and 70.9 (±2.5 g/day) in 2013-14 and no significant difference was observed for levels of intake over time (*p* = 0.247). Furthermore, in 2013–2014, approximately 27% of adults consumed pulses at least once in the 2 days of intake reported. The amount of pulses consumed on pulse consuming days was higher at 117.8 ± 3.5 g/day (data not shown) than intakes based on the average of 2 days.

Demographic data showed that age, sex, ethnicity and education differed by consumer status (Table 1). Pulse consumers were more likely to be male and between the ages of 31 and 70. Both older adults (age >70 years) and younger adults (age 19–31 years) were less likely to be consumers. Mexican-Americans and other Hispanics were more likely to be pulse consumers than other ethnic groups. Those respondents with a greater than high school education were also more likely to be consumers than those with less education.

At all levels (quartiles) of intake, pulse consumers had higher (*p* < 0.01) energy adjusted intakes of fiber, folate and magnesium (Table 2). Potassium was higher at intakes above 40 g/day (2nd quartile). For other nutrients such as choline, iron, zinc and phosphorus, significantly higher energy adjusted intakes were observed in the third and fourth quartiles (those consuming ≥69.4 ± 1.01 g/day). Fat intakes were lower in the third and fourth quartiles. Among pulse consumers, 26% of total folate and fiber intakes were from pulses (data not shown). Pulses also contributed >10% of total intakes of protein, thiamin, iron, magnesium, phosphorus, and zinc.

## 4. Discussion

Based on a nationally representative sample of adults’ ≥19 years, pulse intakes for the total population and for pulse consumers have remained relatively stable from 2003 to 2014. With the exception of the present study, there is limited data evaluating consumption of pulses over time and few studies that examine pulses as a separate subgroup of legumes. Rehm et al. [16] examined trends from 1999–2010 in intake of nuts, seeds and legumes as a subcategory for scoring dietary quality according to the American Heart Association’s 2020 Strategic Impact Goals. Results showed an increase for the subcategory, but was attributable to increases in nuts and seeds and not legumes. In a more recent study using NHANES 2011–2014, 2-day median intake of pulses were the same across the 4-year period [17]. The only other evidence that intakes of legumes or pulses have changed very little are general reports on WWEIA, and NHANES data that compared total vegetable intake, which includes legumes, in 2003–2004 and 2015–2016 [18].

There is also very little comparable data available on trends in global intakes specific to pulses. Data are country specific and inconsistent due to variability in economic factors (e.g., income, cost of pulses, production of animal sources of protein) that impact consumption of pulses [19]. The average level of global consumption as reported by the Food and Agriculture Organization is 21 g per capita per day and has not changed in three decades. These data, however, are based on per capita supply or the availability of pulses for use as food as an estimate of the average consumption. While not directly comparable, the data do offer some insight into overall consumption levels. The highest level of consumption based on per capita supply is Latin America and the Caribbean at 34 g per capita per day. South Asia and sub-Saharan Africa also have higher levels of consumption (33 g) than all other countries including North America with a level of consumption reported at 11 g [19]. Per capita daily pulse consumption based on our intake data was about 19 g. Depending on pulse type, 1/2 cup of cooked pulses weighs between 82 and 100 g and to meet the current 1.5 cup weekly recommendation according to the dietary guidelines [11], an individual would have to consume 246–300 g of cooked pulses per week and per capita daily pulse consumption would have to be 35–43 g per day which is considerably higher than the per capital daily consumption reported in 2013–2014.

The amount of pulses consumed by pulse consumers in this study (~71 g/day) is lower than previously reported data on pulses from NHANES 1999–2002 which was based on a single day of intake (~122 g/day) [2]. Similarly, in a Canadian study over a similar time period, pulse intake from a single day was 113 g/day [20]. The differences among these studies are also quite evident in the distribution of pulse intake across the quartiles and may be explained by differences in the analysis of 1 day of intake in the earlier study [2] vs. 2 days of intake in the current study. In this study, pulse intake was calculated as the average across 2 days with a pulse consumer defined as consuming pulses on at least 1 of 2 days. Unless pulses are consumed on both days, the average consumption across two days would be lower compared to when pulses are only consumed on 1 day.

There are also notable differences in the prevalence of pulse consumption reported among the previous survey published in 2009 [2], a Canadian study [20] and the current analysis. This current study found that 27% of U.S. adults reported consuming pulses on at least one day of their reported 2 days of intake and was considerably higher than the previous single day estimate of 7.9% [2] in the U.S. and 13% in Canada [20] These discrepancies were likely influenced by the number of days of intake examined and the frequency of pulse consumption in their respective populations. For episodically consumed foods (i.e., foods not consumed every day), more days of intake likely captured more respondents who were pulse consumers. For this reason, the use of both days may be more useful for describing population-based consumption patterns of episodically consumed foods [21]. Other factors such as sample size and ethnicity may also explain differences in prevalence and pulse intake estimates among these studies. For example, the Canadian study had twice the sample size than that of the U.S. study [2] with a high consumption of mung beans attributable to a high proportion of Asians in the sample [20]. Several studies have reported on the increases in nutrient density or diet quality in pulse or legume consumers [2,10,19,22] including some nutrients of concern, such as fiber, potassium, choline and magnesium as identified by the Dietary Guidelines Committee [23]. The data from this study support findings found in the earlier study on reported adult intakes from NHANES 1999–2002 for pulse consumers [2]. Nutrient intakes and energy adjusted nutrient intakes or nutrient density (amount of nutrient per 1000 kcal) of several key nutrients including fiber, iron, magnesium, zinc, selenium, phosphorus, potassium, folate, and choline were higher in consumers than non-consumers. The most pronounced improvements in nutrient intakes were seen at average intakes of greater than 69 g (<1/2 cup) of pulses across 2 days of intake. Fiber intakes were significantly higher in pulse consumers compared to non-consumers even at the lowest level of intake (~17 g/day). Increases in energy and percent of calories from carbohydrate were greatest at the highest levels of pulse consumption (i.e., 3rd and 4th quartile) and percent calories from fat was lower in pulse consumers than non-consumers. This finding, in part, may be explained by the sources of pulse containing foods. Similar to what was previously reported by Mitchell et al. [2], food sources of pulses in the U.S. are largely from dry beans with a prevalence of 25% of the population consuming dry beans in the U.S. population over 2 days (data not shown). Dry beans were consumed by themselves, as the main ingredient in a side dish (e.g., baked beans and other canned dry beans), or as dry beans consumed in mixed dishes (e.g., burritos, beans and rice, chili, and soup). Hummus or chickpeas represented approximately 2% of all pulse consumers. The prevalence of other sources (lentils and dry peas) consumed were too low (<1% of all pulse consumers) to accurately estimate consumption.

Understanding the limitations of the dietary exposure literature relative to pulse consumption is important for the future direction of the research. Errors in self-reported dietary intake are inherent in all dietary exposure research and have been well documented in the literature including memory issues, under and over reporting of food intake and errors in portion size estimation [21]. NHANES and other populations-based dietary surveys capture cross-sectional dietary intakes and may not represent longer-term usual intake. These data are usually from a single day or 2 days of intake and are difficult to translate into consumption patterns over the course of week; which is the time frame used to provide dietary guidance in the U.S. This is particularly true for episodically consumed foods. Even with these efforts to characterize pulse consumers (pulse consumption on at least 1 day of intake) by disaggregating food sources that contain small quantities of pulses, there are likely sample respondents that consume pulses less frequently that were classified as non-consumers (no pulses consumed on either day of intake). Therefore, in this study and other cross-sectional surveys, the data may not be a true comparison of consumers and non-consumers, but rather an accounting of pulse intake and the impact on nutrient intakes on days when pulses were consumed.

The Dietary Guidelines recommend healthier dietary patterns by focusing on variety, nutrient density, and amounts of foods to stay within calorie limits. In the 2015–2020 Dietary Guidelines for Americans, the recommendations for the category of legumes (dry beans and peas) were updated so that they could be counted as both a vegetable and as a protein food [23]. Previous guidelines have often times confused consumers and health professionals by ambiguous terminology or by counting legumes in one of two groups (vegetable and meat or protein foods) but not both. Thus, the ability to count pulses as both a vegetable and protein, can better help consumers remain within caloric limits of the dietary patterns. In the most recent iteration of the Dietary Guidelines for Americans 2020–2025, the terminology for the vegetable subgroup legumes (dry beans and peas) has been replaced with beans, peas and lentils [11]. Further clarification has been added about the subcategory, beans, peas and lentils, including that they are also known as pulses. This change removes some of the ambiguity around the broader category of legumes that may have previously confused consumers.

There are many perceived barriers to consuming pulses including difficulty and time-consuming aspects of cooking dry forms, gastrointestinal discomfort, cultural and traditional influences, sensory issues and lack of diverse food choices containing pulses [24]. A strategy suggested by the Dietary Guidelines [20] is to use legumes or nuts and seeds in mixed dishes as a substitute for other foods that are often overconsumed and/or higher in saturated fat, sodium or refined carbohydrate. This shift to a more plant-based diet is sometimes referred to as a flexitarian approach to eating, is becoming increasingly popular because of its perceived health and sustainability benefit by encouraging plant-based protein sources such as pulses while allowing for some meat in moderation. In a recent study, food pattern or menu modeling by substituting less healthy dips and spreads with hummus showed that this simple substitution can reduce energy intake, increase protein intake, and more easily facilitate an increase in legume or vegetable recommendations [10]. Additionally, pulses contain complex carbohydrate and resistant starch and can easily replace foods that are a significant source of refined carbohydrates. With double the amount of protein found in wheat and about three times the amount of protein in rice, pulses may be a more nutrient dense alternative.

The unique composition of pulses makes them well suited for incorporation into a multitude of products. For example, flours made from pulses have been incorporated into snack foods, bread products, meat products, pastas, cereals, soups and beverages. More innovative food technologies could play a role in overcoming hurdles to increase pulse consumption making pulse-based food sources more palatable and nutritious. Future research from national food intake surveys that quantify the impact of pulse ingredients as additions and/or replacements in multi-component foods will be important to further capture the impact of pulses on nutrient intakes and to develop other strategies to increase intake.

As dietary guidance continues to evolve, it is also essential that future research aims to understand the effect of pulses on nutrient and food group intakes in a dynamic marketplace. In this study, trends in intakes over a 10-year period from 2003–2014 have remained stable. With the growing interest in a more sustainable food supply, and more knowledge about plant-based diets and healthier dietary patterns, increases in pulse intake could be on the horizon. Intakes of important nutrients were significantly higher in adults on days when pulses were consumed suggesting that diet quality could be improved by consuming pulses more frequently. Moving the U.S. diet towards a more sustainable and healthier dietary pattern that includes more pulses could have a significant public health impact.

## Figures and Tables

**Table 1:** Demographic characteristics of pulse ^a^ consumers and non-consumers based on 2-day intakes from NHANES, 2013–2014.

Characteristic	Consumers (*n* = 1325)	Non-Consumers (*n* = 3270)	*p* Value ^b^
	-------------------%------------------		
**Age (years)**			0.0318
19–30	19.5	21.8	
31–50	38.4	33.8	
51–70	32.5	33.2	
>70	9.6	11.3	
**Sex**			0.0046
Male	50.7	45.7	
Female	49.2	54.3	
**Ethnicity**			<0.0001
Non-Hispanic White	59.7	70.1	
Non-Hispanic Black	8.8	11.7	
Non-Hispanic Asian	4.9	4.4	
Mexican American	15.2	6.3	
Other Hispanic	8.1	4.7	
Other (incl. multiracial)	3.1	2.8	
**Education ^c^**			<0.0001
<High School	17.4	12.1	
High School	18.5	22.3	
>High School	63.0	63.9	

^a^ Pulses include dry beans, peas, chickpeas and lentils; ^b^ χ^2^ test for significance at *p* < 0.05 for the difference between consumers and non-consumers by demographic characteristic; ^c^ 1.1% of consumers and 1.7% of non-consumers did not report education.

**Table 2:** Pulse amount, energy and nutrient intake for non-consumers and by quartile of pulse consumers based on 2-day dietary intakes from the National Health and Nutrition Examination Survey (NHANES), 2013–2014.

	Non-Consumers (*n* = 3270)	Pulse Consumers (*n* = 1325)			
		Quartile 1 (*n* = 294)	Quartile 2 (*n* = 336)	Quartile 3 (*n* = 322)	Quartile 4 (*n* = 373)
	*----------------------------------Mean ± SE----------------------------------*				
**Pulse intake (g/day)**	------	17.1 ± 0.5	40.7 ± 0.4	69.4 ± 0.6	156.2 ± 4.2
**Energy (kcal/day)**	2029 ± 14	2014 ± 39	2101 ± 40	2333 ± 50 *	2486 ± 50 *
**Macronutrients**					
Protein (g/day)	81.0 ± 0.6	81.6 ± 1.8	84.5 ± 2.0	90.7 ± 2.4 *	100.1 ± 2.1 *
Protein (% kcal)	16.4 ± 0.1	16.5 ± 0.2	16.2 ± 0.3	15.9 ± 0.2	16.4 ± 0.2 *
Carbohydrates (g/day)	240.3 ± 1.8	229.2 ± 4.7	251.7 ± 5.0 *	284.1 ± 6.3 *	308.3 ± 6.2 *
Carbohydrates (% kcal)	47.6 ± 0.2	46.1 ± 0.5	48.5 ± 0.5	49.3 ± 0.5	50.2 ± 0.4 *
Total Fat (g/day)	78.8 ± 0.6	79.4 ± 2.0	80.6 ± 1.9	87.9 ± 2.6	89.7 ± 2.2 *
Total Fat (% kcal)	34.6 ± 0.1	35.1 ± 0.5	34.0 ± 0.3	33.3 ± 0.4 *	32.0 ± 0.3 *
Fiber, total dietary (g/day)	15.4 ± 0.1	18.1 ± 0.4 *	18.5 ± 0.4 *	23.6 ± 0.7 *	28.6 ± 0.6 *
Fiber, total dietary (g/1000 kcal)	7.8 ± 0.1	9.4 ± 0.2 *	9.3 ± 0.2 *	10.6 ± 0.2 *	12.2 ± 0.2 *
**Micronutrients**					
Calcium (mg/day)	940 ± 9	1050 ± 36	970 ± 26 *	1096 ± 28 *	1196 ± 31 *
Magnesium (mg/day)	285 ± 2	318 ± 6 *	310 ± 7 *	374 ± 12 *	383 ± 7 *
Iron (mg/day)	14.2 ± 0.1	14.5 ± 0.4	14.6 ± 0.3 *	17.4 ± 0.5 *	18.5 ± 0.4 *
Phosphorus (mg/day)	1341 ± 10	1409 ± 32	1405 ±31	1559 ± 39 *	1691 ± 34 *
Selenium (mcg/day)	116.0 ± 0.9	109.5 ± 2.4	116.1 ± 2.8	126.0 ± 4.2	131.3 ± 3.1 *
Zinc (mg/day)	10.9 ± 0.1	11.2 ± 0.3	11.5 ± 0.3 *	13.0 ± 0.4 *	13.1 ± 0.3 *
Potassium (mg/day)	2537 ± 18	2655 ± 49	2701 ± 54 *	3191 ± 69 *	3271 ± 58 *
Folate, food (mcg/day)	204 ± 2	239 ± 6 *	227 ± 5 *	282 ± 0.8 *	357 ± 10 *
Niacin (mg/day)	25.8 ± 0.2	26.0 ± 0.7	25.3 ± 0.9	28.0 ± 1.2	29.3 ± 0.7 *
Riboflavin (mg/day)	2.1 ± 0.0	2.3 ± 0.1	2.1 ± 0.1	2.3 ± 0.1 *	2.3 ± 0.1 *
Thiamin (mg/day)	1.6 ± 0.0	1.5 ± 0.0	1.6 ± 0.0	1.9 ± 0.1 *	2.0 ± 0.1 *
Choline, total (mg/day)	324 ± 3	328 ± 8	329 ± 8	375 ± 11 *	413 ± 10 *
**Micronutrient Density**					
Calcium (mg/1000 kcal)	475 ± 3	519 ± 12	467 ± 9	484 ± 10	448 ± 8
Magnesium (mg/1000 kcal)	146 ± 1	164 ± 3 *	152 ± 3 *	164 ± 3 *	161 ± 2 *
Iron (mg/1000 kcal)	7.1 ± 0.0	7.5 ± 0.2 *	7.2 ± 0.1	7.6 ± 0.1 *	7.7 ± 0.1 *
Phosphorus (mg/1000 kcal)	672 ± 3	704 ± 9	673 ± 8	681 ± 9 *	693 ± 7 *
Selenium (mcg/1000 kcal)	58.5 ± 0.3	55.8 ± 0.9	56.3 ± 1.1 *	54.2 ± 0.9 *	53.3 ± 0.8 *
Zinc (mg/1000 kcal)	5.4 ± 0.0	5.7 ± 0.1	5.5 ± 0.1	5.7 ± 0.1 *	5.4 ± 0.1 *
Potassium (mg/1000 kcal)	1296 ± 7	1370 ± 22	1330 ± 20 *	1357 ± 22 *	1345 ± 20 *
Folate, DFE ** (mcg/1000 kcal)	105 ± 1	124 ± 4 *	114 ± 3 *	127 ± 4.0 *	151 ± 4.0 *
Niacin (mg/1000 kcal)	13.0 ± 0.1	13.4 ± 0.3	12.1 ± 0.3 *	12.1 ± 0.2	11.9 ± 0.2 *
Riboflavin (mg/1000 kcal)	1.1 ± 0.0	1.2 ± 0.0	1.0 ± 0.0	1.1 ± 0.0	0.9 ± 0.0 *
Thiamin (mg/1000 kcal)	0.8 ± 0.0	0.8 ± 0.0	0.8 ± 0.0	0.8 ± 0.0	0.8 ± 0.0 *
Choline (mg/1000 kcal)	163 ± 1	166 ± 3	160 ± 3.0	165 ± 3.0 *	169 ± 3.0 *

* Significantly (*p* < 0.01) different from non-consumers; ** DFE = dietary folate equivalents

## Data Availability

All data used for this study is from publicly available data sets available at the Centers for Disease Control and Prevention (CDC). National Center for Health Statistics (NCHS). National Health and Nutrition Examination Survey. https://wwwn.cdc.gov/nchs/nhanes/ (accessed on 15 August 2019).
